# Judgments of learning impair rule-based discovery

**DOI:** 10.3758/s13421-025-01737-6

**Published:** 2025-06-04

**Authors:** Kit S. Double, Dominic Tran, Micah B. Goldwater

**Affiliations:** School of Psychology, The University of Sydney, Sydney, NSW Australia

**Keywords:** Reactivity, Category learning, Rule-based discovery, Relational learning, Judgments of learning, Memorization, Knowledge transfer

## Abstract

Eliciting judgments of learning (JOLs) from participants has been shown to enhance memory recall on paired-associates tasks in some contexts, a *reactivity effect*. However, little is known about the effect of JOLs on forms of learning where the task requires generalizing beyond the training set, such as in category learning. Here, we explore the effect of JOLs on relational rule discovery using a categorization paradigm. In Experiments 1 and 2, where both a relational rule and visual stimulus features can be effectively used for categorization, we show that JOLs impair rule discovery but do not affect memorization of visual features. In Experiment 3, we modified the task such that only a relational rule could be used to categorize stimuli and observed no evidence of reactivity. We explain these findings using a conservative strategy-shift account of reactivity, which proposes that eliciting JOLs causes participants to shift strategies to utilize the more obviously rewarded strategy. Specifically, when participants have multiple viable strategies, JOLs shift participants’ categorization strategy away from rule discovery and instead encourage a more obvious strategy based on memorization of visual features.

**Supplementary Information:**

The online version contains supplementary material available at 10.3758/s13421-025-01737-6.

## Introduction

Metacognition plays a central role in our ability to learn (Doyle & Hourihan, 2016; Veenman et al., 2006; Zimmerman & Moylan, 2009). It is generally believed that our metacognitive judgments directly affect our choice of how and what to learn (Metcalfe & Kornell, 2005). For instance, while studying for an upcoming exam a student may make several metacognitive judgments (e.g., have I learnt this material well enough?) which might prompt various changes in learning (e.g., I need to study this material further). Researchers interested in metacognition typically assess metacognition by eliciting self-report ratings from participants. A judgment of learning (JOL), for example, is a rating whereby participants indicate how likely they are to recall the studied material on a future test. JOLs have been shown to predict actual learning and have been useful in helping unpack the process involved in metacognition (Koriat, 2007). However, recent evidence suggests that merely eliciting metacognitive judgments can affect learning, a so-called *reactivity effect* (Double et al., 2018; Janes et al., 2018; Mitchum et al., 2016; Soderstrom et al., 2015).

Many studies have shown that, under certain conditions, eliciting JOLs from participants while they are learning enhances later recall of the material (Ariel et al., 2021; Double & Birney, 2019b; Double et al., 2018; Halamish & Undorf, 2023; Janes et al., 2018; Li et al., 2021; Maxwell & Huff, 2022; Mitchum et al., 2016; Myers et al., 2020; Rivers et al., 2021; Senkova & Otani, 2021; Shi et al., 2023; Soderstrom et al., 2015; Tauber & Witherby, 2019; Tekin & Roediger III, 2020; Witherby & Tauber, 2017; Yang et al., 2015; Zhao et al., 2022), while others have shown evidence that eliciting JOLs can impair later recall (Double, 2023; Mitchum et al., 2016; Undorf et al., 2024). The effects have largely been established within a paired-associates paradigm where participants first study a set of cue-target word-pairs (e.g., cat-lamp) before later being tested on their recall of the targets when given the cues. The most common outcome of these studies is to observe positive reactivity (a memorial benefit) of JOLs for semantically related cue-target pairs (e.g., dog-bone) and no reactivity for semantically unrelated cue-target pairs (e.g., dog-potato).

## Relational category learning

While reactivity has been shown using experimental paradigms that require participants to memorize a series of stimuli, less is known about reactivity when the task requires generalizing beyond the training set, such as in category learning. Relational categories are stimulus categories defined not by the intrinsic properties of the objects themselves but by the relations among those objects (Goldwater & Schalk, 2016). Intrinsic (feature-based) cues refer to attributes that are literally part of the object – for example, a bird’s plumage color, the number of wheels on a vehicle, or the atomic weight of an element – each of which can legitimately serve as a basis for grouping items that share that attribute. By contrast, relational cues depend on how an object stands in relation to something else – for instance, whether one line is longer than another, whether a figure is to the left or right of a reference point, or whether one animal is chasing another. Because relational cues abstract away from surface form, they often support much broader generalization than intrinsic features; a “hunter,” for example, can be a lion, a human, or even a corporate start-up, so long as it bears the relation of pursuing a target.

Relational concepts are common in our everyday lexicon, often derived from social roles (e.g., hero, victim, guest, conduit), but can be based on any kind of relation, (e.g., classifying a substance as a poison or a drug is not based on intrinsic molecular features, but the effects it has on others). Goldwater and Schalk (2016) argue that relational concepts are central to formal education, particularly in STEM fields. The goal of concept learning in formal education is to support students to apply their knowledge to new contexts and problems that may differ on the surface but share common underlying structure. For example, with the right concept-driven instruction, even young children can apply concepts of *ecological competitio*n and *selective reproduction* beyond their learning contexts to explain the development of novel species (Emmons, Lees, & Keleman, 2018). Showing the importance of categorization, analyses of failures to solve novel math problems show that errors are often based in erroneous problem categorization, where learners misapply solution strategies for one problem category to examples of a different category (Rohrer et al., 2014). Because of the ubiquity and importance of relational concepts, Goldwater and Schalk (2016) have argued that relational category-learning tasks offer a useful laboratory model for formal concept learning, allowing researchers to develop formal theories of learning and form a testing ground for novel learning interventions.

While some cognitive processes are involved in all forms of learning, relational category learning requires additional processes compared to memorization and feature-based learning (Goldwater & Schalk, 2016), as, for example, shown by model-based analyses of functional magnetic resonance imaging (fMRI) data comparing feature-based and relational category learning (Davis et al., 2017). For instance, relational learning requires representing the meaning of objects and reasoning within and across stimuli. This taps into elaborative learning processes, such as analogical reasoning (Kurtz et al., 2013), which are important for our most valued educational outcomes such as conceptualizing the natural and social world in terms of abstract yet coherent schemas (Resnick, 2010).

Relational category learning is often contrasted with feature-based category learning. Feature-based learning involves participants learning about how the intrinsic features of stimuli relate to category membership (Goldwater et al., 2018). Because relational categories are defined by relational patterns orthogonal to the intrinsic properties of stimuli, they are crucial to promote “far transfer,” which is the application of prior knowledge to new contexts that share few surface similarities with the initial context of learning (Goldwater & Schalk, 2016). Notably, real-world learning typically involves elements of both rule-learning and memorization, with educators often designing materials where the deep structure and surface content are correlated (Goldwater & Schalk, 2016). For example, analyses of math textbooks show that the superficial content of math problems is correlated with the structure of the math problem (Bassok et al., 1998). However, if exemplars that dissociate deep structure and surface content are never presented, this can limit relational abstraction, tying structure understanding to the specific content of learning examples. In laboratory studies that correlate surface features with deeper relations during learning, the ability to generalize based on either features or relations seem to trade-off with each other (i.e., they are negatively correlated), suggesting they are tapping into distinct learning strategies, or at least competing attentional foci (Goldwater et al., 2018; McDaniel et al., 2018).

## Reactivity and relational discovery

To generate hypotheses about the effect of JOLs on relational rule discovery we draw from an existing theory of reactivity, *the changed goal hypothesis*, and integrate it with recent findings that eliciting confidence ratings produces more conservative responding (Li et al., 2023)*.* Across a range of cognitive tasks, eliciting metacognitive judgments has been shown to make learners more cautious and accuracy-oriented: they adopt higher decision thresholds, attempt fewer items when speed is optional, and invest effort where success feels most certain (Double & Birney, 2018; Li et al., 2023). For example, drift–diffusion modelling has shown this conservative shift as an elevation of the evidence criterion required before committing to a response when confidence ratings are elicited (Li et al., 2023). While the surface expression of a conservative-shift in strategy differs with task demands, the functional outcome is the same: learners gravitate toward the strategy that maximizes immediate, demonstrable performance, even if that choice sacrifices deeper mastery or far transfer. This mechanism dovetails with the metamemory changed-goal hypothesis, which holds that JOLs can redirect study time from difficult to easy items once individuals realize perfect performance is unattainable (Mitchum et al., 2016). For instance, Mitchum et al. (2016) found that, when JOLs were elicited, participants spent longer studying the easier semantically related word pairs and less time studying the more difficult semantically unrelated word pairs. We therefore treat both views as facets of a unified conservative strategy-shift account: metacognitive prompts repeatedly spotlight prospective or retrospective success, nudging learners to favor the most readily rewarded option – memorizing salient features, slowing responses, or skipping hard items – at the cost of more abstract, rule-based learning.

A further finding within JOL reactivity research that is relevant to the current context is the idea that JOLs induce item-specific processing (Kaya & Mulligan, 2024; Senkova & Otani, 2021; Zhao et al., 2023a, 2023a, 2023b). Several studies have suggested that eliciting JOLs from participants increases recall of item-specific features at the cost of inter-item processing (Senkova & Otani, 2021; Zhao et al., 2023a). For example, Zhao et al. (2023a) found that JOLs improve recognition of item features while negatively affecting recall of item-order. These findings have led researchers to suggest that JOLs prompt participants to adopt strategies that prioritize item-specific processing (Senkova & Otani, 2021). This focus may consequently disrupt inter-item relational processing, as cognitive resources become disproportionately allocated to individual items, leaving fewer resources available for processing relationships between items. Note, however, that learning to categorize will always require some degree of comparison between stimuli, but if JOLs steer learners toward information that is perceptually salient and easily individuated (i.e., “item-specific”) rather than toward relations that must be abstracted across multiple dimensions or exemplars they are likely to be particularly troublesome in the context of relational rule-discovery.

Recently, three studies have explored the effect of JOLs in a category-learning paradigm. Lee and Ha (2019) looked at whether JOLs could enhance the learning of categories. To do this they had some participants make JOLs during a first category learning phase and some participants restudy the category examples. Participants then all completed a second learning phase without JOLs. They assessed learning by examining performance on novel transfer items but found no evidence that eliciting JOLs facilitated category learning. In a similar design, Wang et al. (2023) found evidence that JOLs enhance learning for a difficult categorization task but not an easy categorization task. While these provide some preliminary evidence about reactivity in a categorization paradigm, both studies used feature-based category learning and we do not yet know how eliciting JOLs will affect participants’ learning of relational rules, the central question asked here.

Moreover, recent work using a category-learning paradigm showed that on-line confidence ratings also undermine rule abstraction. In a large, preregistered sample (N = 710), Double et al. (2025) found that asking participants for a confidence judgment after every response reduced rule-based classification on ambiguous transfer items while leaving overall learning intact. In contrast, they found that a condition making JOLs did not differ from the control group. Although Double et al. (2025) found that JOLs left rule learning unaffected, their study offered learners no salient surface cue that could serve as an expedient alternative to the rule, and the JOL prompt was framed globally (“How well have you learnt…?”). Current conservative-strategy theories predict that JOLs will become disruptive to rule-learning precisely when (a) an easier, feature-based route is available and (b) the learner is reminded – item by item – of impending test performance. Guided by this a priori reasoning, the present experiments introduce a highly salient color cue, manipulate its instructional salience, and employ an all-items accuracy-prediction JOL to test whether these conditions are sufficient to make JOLs, like confidence ratings, bias learners away from relational rule learning. By doing so we aim to specify the boundary conditions of JOL reactivity and reconcile the apparently benign effect observed by Double et al. with the broader conservative strategy-shift account.

Based on these findings from the reactivity literature we generate several hypotheses about the effect of JOLs on relational rule-discovery. Our exploration of the effect of JOLs on relational rule discovery spans two contexts for which we must draw hypotheses. The first (used in Experiments 1 and 2) is when category membership can be predicted by both the surface visual features of the stimuli (although imperfectly) and the relational rule. The second (used in Experiment 3) is when the surface visual features are non-diagnostic of category membership and the only viable way to determine category membership is to discover the relational rule that governs it.

In the first context where both salient visual features and the more subtle relational rule predict category membership, we would expect poorer rule discovery when JOLs are elicited because participants will prioritize the more obvious strategy during training (using the visual features to predict category membership), rather than sacrifice their short-term performance determining whether other, more subtle, relational stimulus dimensions are better predictors of category membership. This idea is also supported by findings that suggest JOLs lead to better recall of item-specific features and poorer recall of inter-item information (Senkova & Otani, 2021; Zhao et al., 2023a, 2023b). However, in the second context where only the relational rule is diagnostic, we would hypothesize that JOLs should not affect rule learning. According to the conservative strategy-shift account, JOLs only affect performance when participants can strategically shift their resources (e.g., study time). In the context where only the relational rule governs category membership, participants have no alternative effective strategy to perform above chance during training, and must discover the relational rule. Thus, we do not expect that participants in the JOL condition will differ significantly from the control group in terms of their resource allocation because there is simply no viable alternative strategy in the second context.

## Experiment 1

### Method

#### Participants

The university human ethics committee approved the study and informed consent was obtained. Participants were recruited online from Prolific Academic. Participation was limited to participants residing in the USA or UK. Sample size was determined using a power analysis in G*power for a repeated-measures ANOVA with a within-between interaction (two groups, four measurements, power = 0.8, r = 0.5). The power analysis indicated that a minimum sample of 62 was required to detect a medium effect (f = 0.15). Sixty-three participants (49.2% female; M_age_ = 36.45 years, SD = 12.66 years) were recruited from Prolific Academic. Participants were randomly allocated to either a JOL condition (n = 30) or a No-JOL condition (n = 33).

#### Materials

**Fig. 1:**
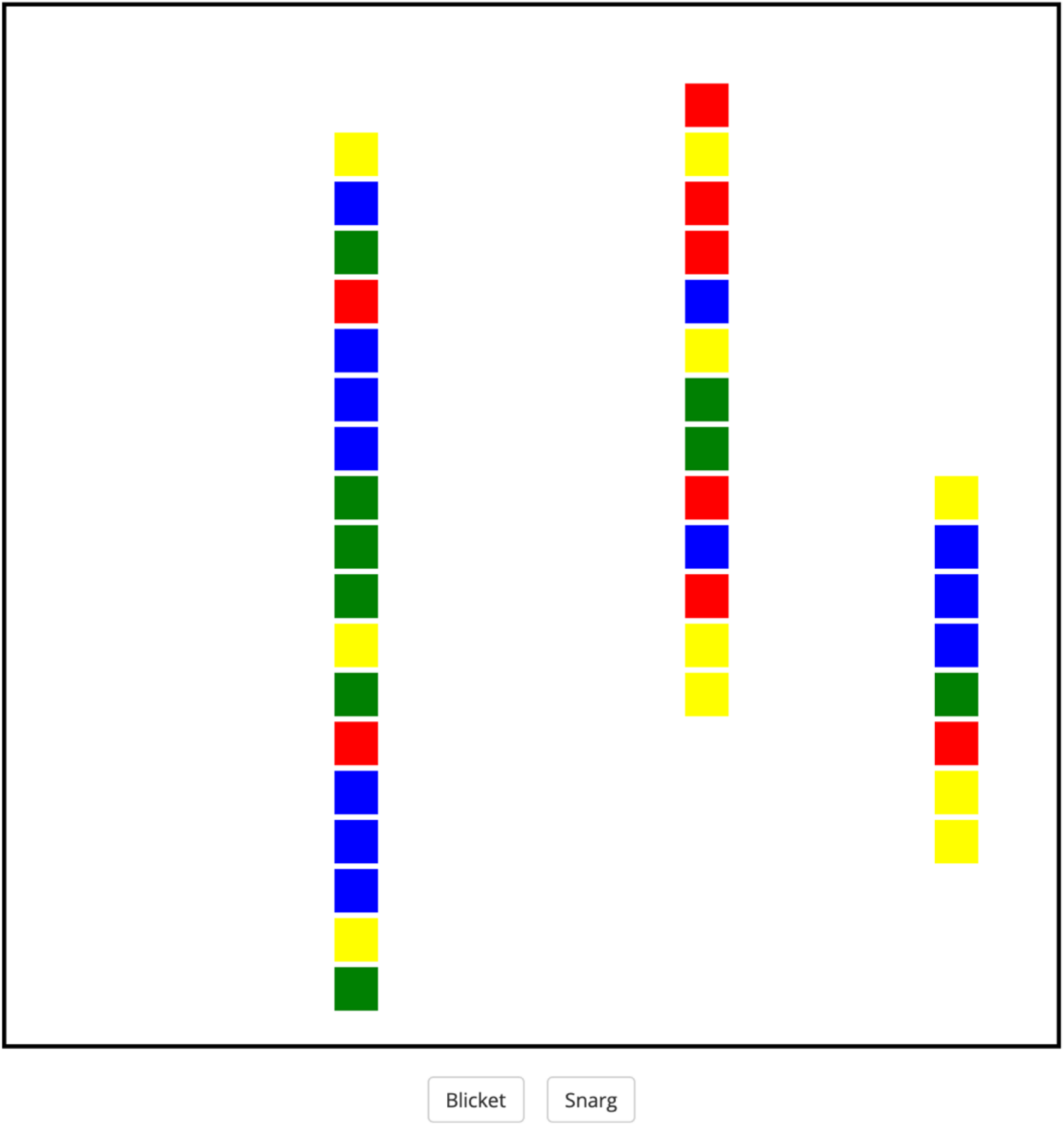
An example of training trial. Stimuli are comprised of three vertical lines each comprising a series of colored squares

The experiment was programmed in jsPsych. The categorization task was based on the task used in Goldwater et al. (2018). It required participants to categorize geometric configurations of three lines into either “blickets” or “snargs.” As shown in Fig. 1, stimuli consisted of a display with three vertical lines composed of colored squares. Each square was 30 px wide and was shown on a visual display comprising 400 potential locations. For each stimulus, squares were shown such that the three lines were randomly placed across the display, with random horizontal and vertical positioning. The length of each line was randomly selected to be between three and 18 squares. Each line differed in length from the other two lines by at least one square.

Stimulus category was defined by a relational rule, the arrangement of the three lines. For one category (e.g., blickets), the three lines were arranged monotonically based on length (either increasing or decreasing in length). For the alternate category (e.g., snargs), the three lines were arranged non-monotonically across the display.

Stimulus category also probabilistically defined the visual features (i.e., color) of each stimulus. The colors of the squares were sampled from a probability distribution (red, green, yellow, blue) which differed between categories. Each category had two colors that were dominant (each 30% of squares, on average) and two colors that were non-dominant (each 20% of squares, on average). The particular colors associated with each category were randomized for each participant. For example, if the monotonic category had red/green dominance for a given participant, and a given stimulus was monotonic, the constituent squares were sampled from a distribution that was 30% red, 30% green, 20% blue, 20% yellow.

#### Procedure

Participants were provided with instructions describing the categorization task and told to learn to distinguish between categories based on the feedback provided. Participants then completed six training blocks of 12 trials. Participants could rest between each block. On each of the training trials, participants indicated whether they believed the stimulus was a “blicket” or “snarg” by pressing one of two buttons with the mouse. Participants had a maximum of 8 s to respond. After responding, participants saw corrective feedback for 1 s. Each category appeared on 50% of training trials.

After receiving feedback, participants in the No-JOL condition continued to the next trial, while participants in the JOL condition made their JOL. The JOL (*Please rate the likelihood that you will correctly indicate whether items are Blickets or Snargs on a later test*) was presented on a sliding scale from 0 *(not at all)* to 100 *(definitely)*.

After completing the six training blocks, participants completed a test block. The test consisted of four different trial types. *Baseline trials* were programmed using the same parameters used in the training phase. *Featural trials* were programmed using the same parameters, except each of the three lines was the exact same length so that the relational rule could not be used. *Relation trials* were programmed using the same parameters, but the distribution of colors was equal (each of the four colors made up 25% of the squares) so that the feature rule could not be used. Finally, *cross-mapped trials* reversed the mapping of the colors and monotonic relationship, such that the colors that were dominant on monotonic stimuli were now dominant on non-monotonic stimuli and vice versa. Cross-mapped trials are scored such that the response aligned with the relational rule was correct. Participants completed eight of each trial type for a total of 32 test trials. Test trials were untimed.

#### Openness and transparency

These experiments were not preregistered. All data, materials, and analysis code are available via the Open Science Framework (https://osf.io/t4fcr/).

### Results

#### Training data

**Fig. 2:**
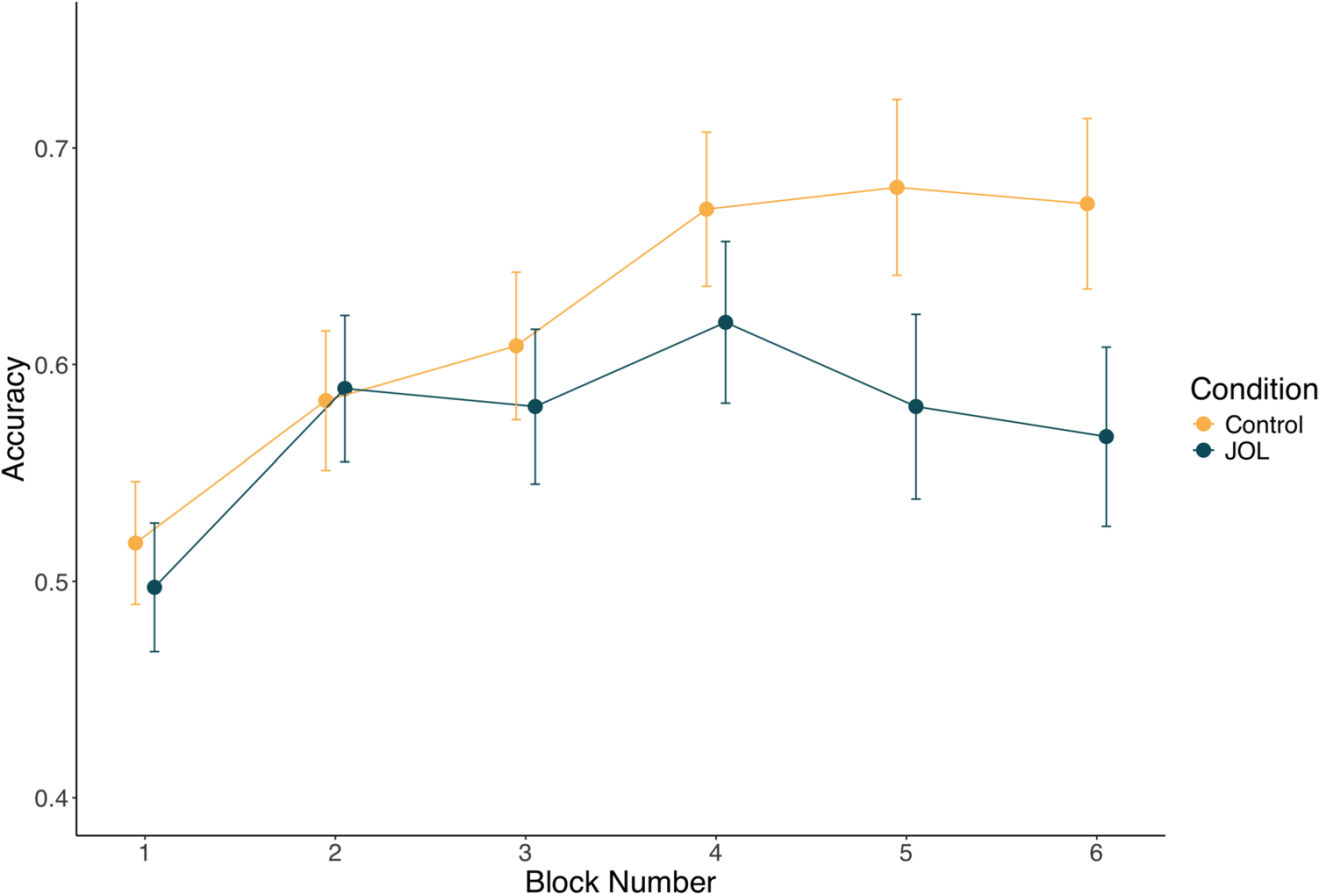
Experiment 1 training data from across the six blocks as a function of experimental condition. Error bars represent one standard error of the mean

Training performance drawn from the six training blocks was subjected to a multilevel model. Block number and experimental condition along with their interaction were entered as predictors, and a random intercept for each participant was included. The model indicated that performance significantly improved across the six training blocks (β = 0.27, *p* < 0.001). The main effect of condition was not significant (β = −0.24, *p* = 0.166), however, the block number X condition interaction was significant (β = −0.18, *p* = 0.018). As shown in Fig. 2, participants in the JOL group tended not to improve across training to the same extent as the control condition.

#### Test data

Test data were subjected to a 2 (condition: JOL vs. Control) × 4 (test type: baseline vs. Feature vs. Relation vs. cross-mapped) mixed between-/within-subjects analysis of variance (ANOVA). The results indicated that there was a significant main effect of test type, *F*(3,183) = 5.24, *p* = 0.002, η_p_^2^ = 0.079. Bonferroni-adjusted pairwise comparisons indicated that performance on Baseline test trials was significantly higher than either Feature test trials, t(61) = 3.72, *p* = 0.003, d = 0.71, or Cross-mapped trials, t(61) = 2.96, *p* = 0.026, d = 0.36. None of the other pairwise comparisons were significant, though performance on Baseline trials was marginally higher than Relation trials, t(61) = 2.58, *p* = 0.074, d = 0.29.

**Fig. 3:**
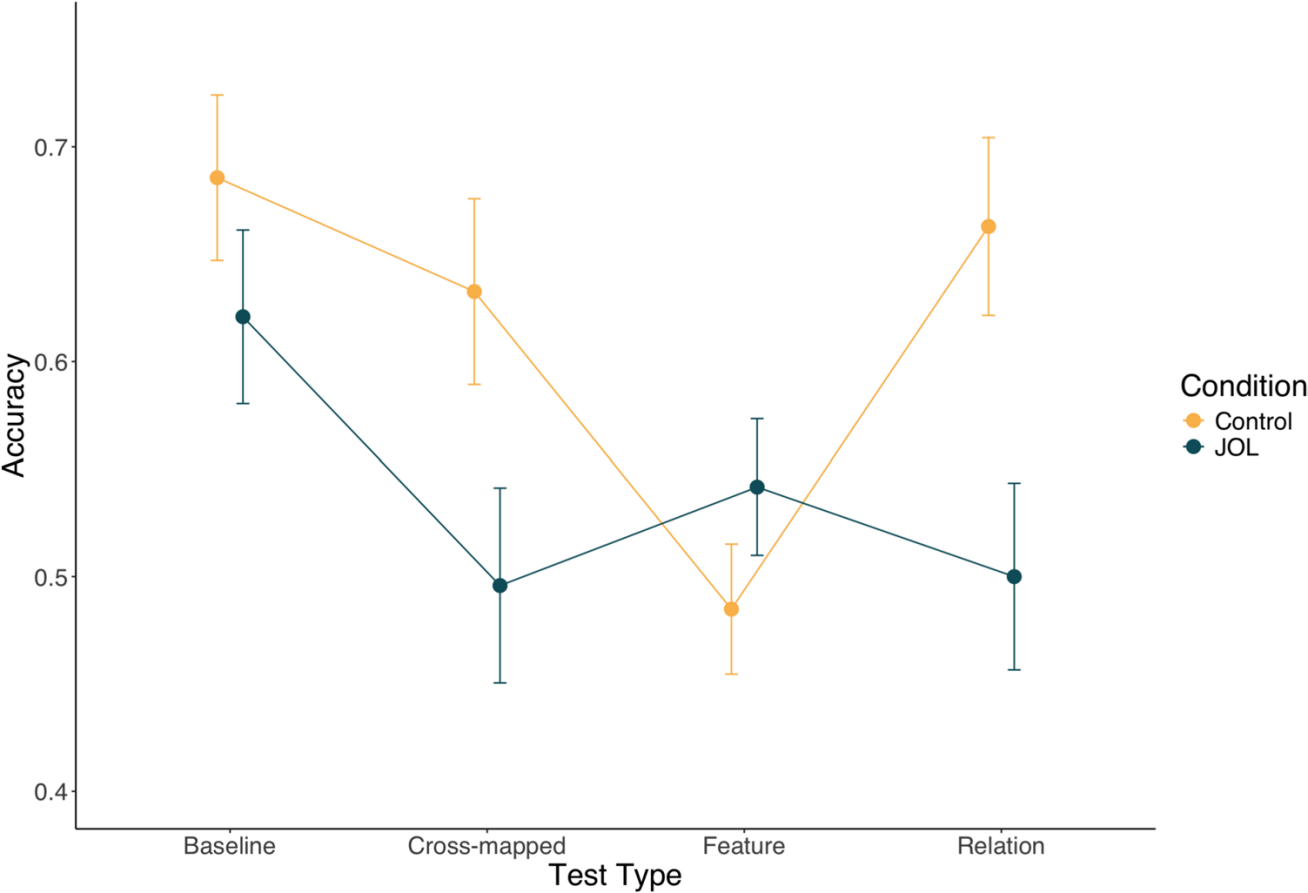
Experiment 1 test data from the four test trial types as a function of experimental condition. Error bars represent one standard error of the mean

The main effect of condition was also significant, *F*(1,61) = 4.83, *p* = 0.032, η_p_^2^ = 0.073, such that participants in the JOL condition tended to perform worse than those in the control condition, averaged across trial types. As shown in Fig. 3, the trial type × condition interaction was also significant, *F*(3,183) = 3.78, *p* = 0.012, η_p_^2^ = 0.058. Bonferroni-adjusted pairwise comparisons indicated that the JOL condition performed significantly worse on Relation test trials, t(61) = 2.71, *p* = 0.009, d = 0.68, and on Cross-mapped test trials, t(61) = 2.18, *p* = 0.033, d = 0.55 (a correct response on Cross-mapped trials aligns with the relational response). There was no significant group difference on Baseline trials, t(61) = 1.16, *p* = 0.251, d = 0.29, nor Feature test trials, t(61) = 1.23, *p* = 0.200, d = −0.33.

Finally, we compared the test performance to chance (50%) for each condition separately. The JOL condition only significantly exceeded chance performance on Baseline trials, t(29) = 3.12, *p* = 0.004, (all other conditions *p* > 0.05). In contrast, the Control condition significantly exceeded chance performance in all test trial types (all p < 0.01) except Featural trials t(32) = 0.52, *p* = 0.600.

These findings support our hypothesis that eliciting JOLs led to a decrease in rule discovery. Participants in the JOL condition showed a selective impairment in both Relation and Cross-mapped trials compared to controls, indicating that, while they were able to learn to categorize the stimuli, they did so using a strategy that focused on the surface features of the stimuli.

## Experiment 2

The findings of Experiment 1 suggest that eliciting JOLs from participants impaired their relational learning. We propose that this is due to a shift in the goals of participants, with participants prioritizing a strategy that is more obvious over the more difficult rule discovery strategy. However, an alternative explanation arises because surface features are more salient than relational structures and thus it may be that JOLs reinforce whatever strategy participants naturally favor. We test this alternative explanation against the conservative strategy-shift account in the following two experiments. Here, in Experiment 2, we deliberately encourage participants to use a particular strategy (either featural or relational). We expect that when participants are instructed to use a particular strategy then they are less likely to react to JOLs by shifting strategies, and we should therefore not see reactivity. Alternatively, if JOLs reinforce whichever strategy participants favor, then JOLs should encourage relational discovery when participants are directed to attend to the relational structure and, similarly, JOLs should encourage featural learning when participants are directed to attend to the surface features. Thus, in terms of relational rule learning, we should observe improvements in rule-learning from JOLs for participants given a relational hint and impaired rule-learning in participants given a featural hint.

### Method

#### Participants

Participants were recruited in the same fashion as Experiment 1. A power analysis was performed in the same way as Experiment 1 with the additional between-subject factor, (six groups, four measurements, power = 0.8, r = 0.5, f = 0.15) indicated a desired sample of 114 participants. To accommodate the additional between-subjects factor we aimed to approximately double our sample size from Experiment 1. 120 participants (50% female; M_age=_ 39.64 years, SD = 14.35 years) were assigned to complete the study either in the JOL condition (n = 57) or the control condition (n = 63). Forty participants were given a relational hint, 40 participants were given a featural hint, and 40 participants were given no hint.

#### Materials and procedure

The method and procedure were the same as Experiment 1 with the following exception. An additional between-person manipulation was included which manipulated whether a hint was included during the instructions phase. Participants in the Feature-Hint condition received the following hint: “Note: here’s a hint for how to solve the task. Pay attention to the colors that appear in each pattern.” Participants in the Relation-Hint condition received the following hint: “Note: here’s a hint for how to solve the task. Compare the lengths of the lines that appear in each pattern.” Finally, a No-Hint condition did not receive a hint and thus was a direct replication of Experiment 1.

### Results

#### Training data

**Fig. 4:**
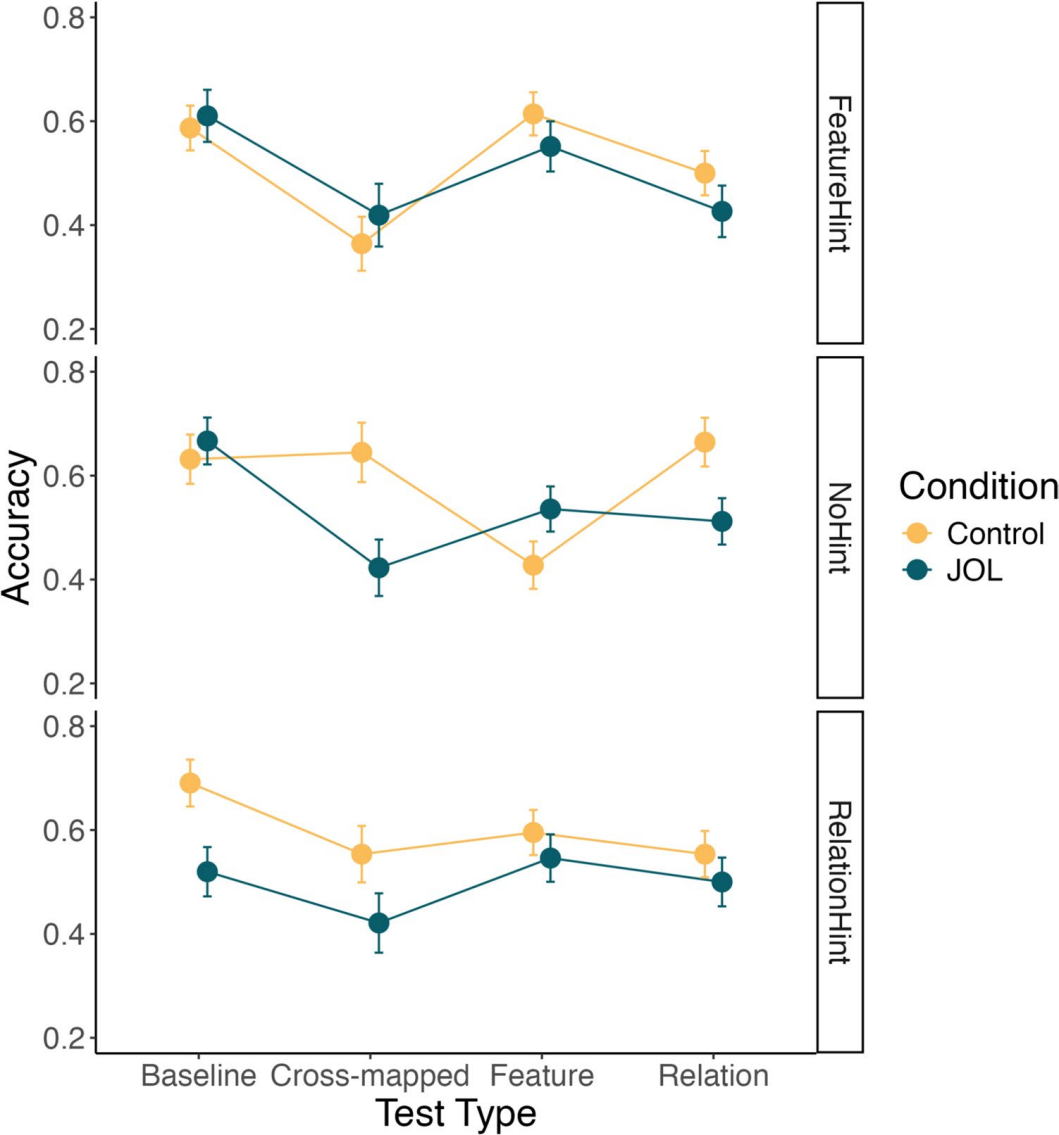
Experiment 2 training data from across the six blocks as a function of judgment and hint condition. Error bars represent one standard error of the mean

Training performance data was subjected to a multilevel model with block number, judgment condition, and hint condition as well as interaction between block and judgment condition and block and hint condition. The model (see Fig. 4) indicated a main effect of block number, such that performance significantly improved across the six training blocks (β = 0.15, *p* = 0.017*)*. In addition, the main effect of judgment condition was significant (β = −0.36, *p* = 0.001), with participants in the JOL condition performing worse over the training blocks. None of the other effects reached significance.

#### Test data

**Fig. 5:**
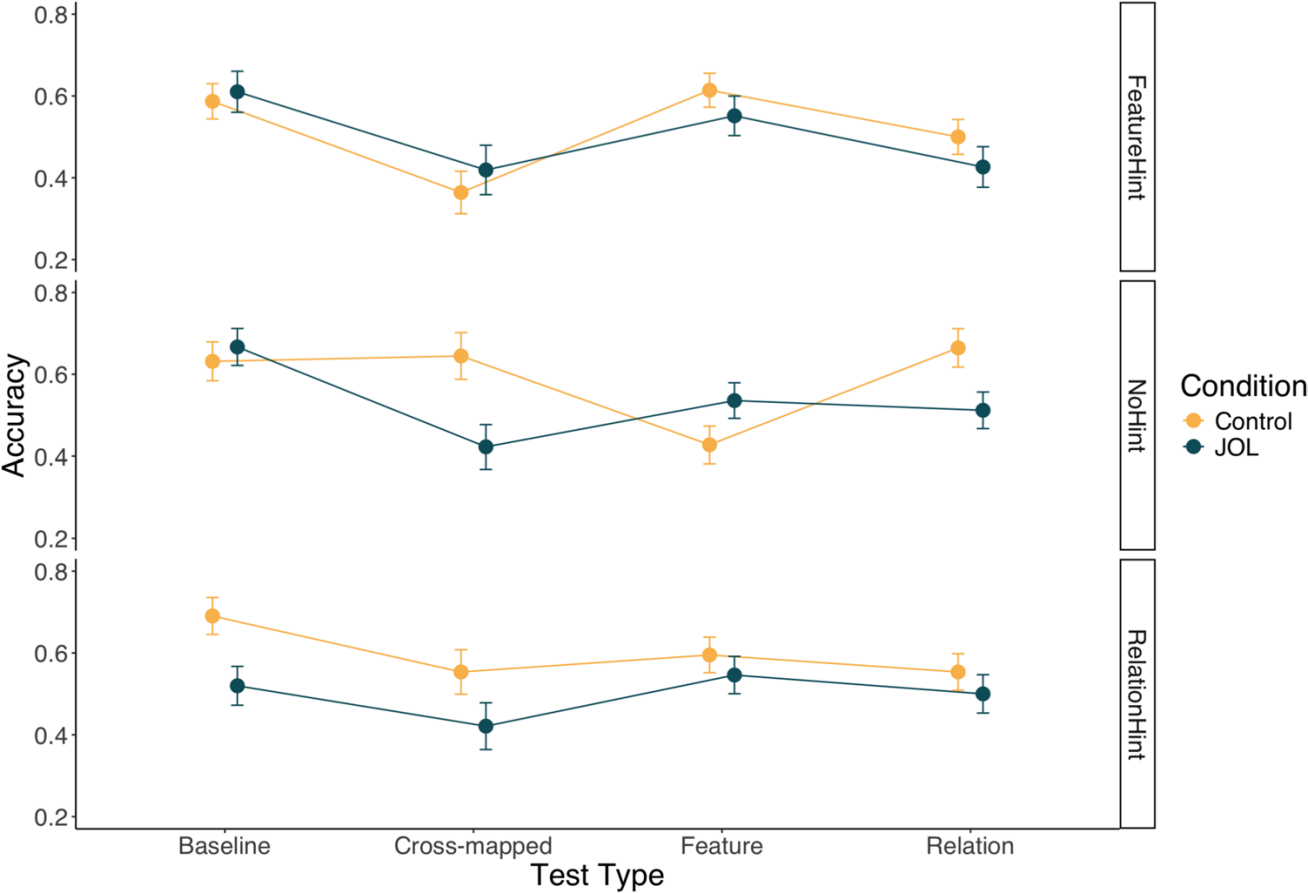
Experiment 2 test data from on the four test trial types as a function of judgment and hint condition. Error bars represent one standard error of the mean

Test data were analyzed using a 2 (judgment condition: JOL vs. control) × 3 (hint condition: no hint vs. feature hint vs. relation hint) × 4 (test type: baseline vs. Feature vs. Relation vs. cross-mapped) mixed between-within subjects ANOVA, as shown in Fig. 5. The main effect of judgment condition was significant, *F*(1,114) = 7.48, *p* = 0.007, η_p_^2^ = 0.062, with participants in the JOL condition doing worse, averaged across the other factors. The main effect of hint condition was not significant averaged over test type and judgment condition, *F*(2,114) = 2.29, *p* = 0.106, η_p_^2^ = 0.039. The results indicated that there was a significant main effect of test type, *F*(3,342) = 9.86, *p* < 0.001, η_p_^2^ = 0.080. Bonferroni-adjusted pairwise comparisons indicated that performance on Baseline test trials was significantly higher than either Feature test trials, t(114) = 3.07, *p* = 0.016, d = 0.34, Cross-mapped trials, t(114) = 5.25, *p* < 0.001, d = 0.63, and Relation trials, t(114) = 4.11, *p* < 0.001, d = 0.43.

The judgment condition × hint condition interaction was not significant, *F*(2,114) = 1.40, *p* = 0.250, η_p_^2^ = 0.024, nor was the judgment condition × test type interaction, *F*(3,342) = 1.48, *p* = 0.218, η_p_^2^ = 0.013. The hint condition × test type interaction was significant, *F*(6,342) = 2.93, *p* = 0.008, η_p_^2^ = 0.049, as was the three-way interaction between hint condition, judgment condition and test type, *F*(6,342) = 2.80, *p* = 0.011, η_p_^2^ = 0.047. Replicating the results of Experiment 1, follow-up pairwise comparisons using Bonferroni corrections comparing the JOL and control judgment conditions indicated that there was a significant negative effect of JOLs for the cross-mapped, t(114) = 2.82, *p* = 0.006, d = 0.83, and relation, t(114) = 2.36, *p* = 0.020, d = 0.66, test trials when no hint was provided. Thus, both Experiment 1 and Experiment 2 provide evidence that JOLs impaired performance on Cross-mapped and Relation test trials. In addition, the JOL condition performed significantly worse on baseline test trials when a relation hint was provided, t(114) = 2.61, *p* = 0.010, d = 0.80. We did not observe significant reactivity on baseline trials in any of the other hint conditions. The effect of JOLs on baseline test trials in the relational hint condition and training performance more generally across conditions may indicate that JOLs hampered learning more broadly than in Experiment 1. We discuss this finding in greater detail in the *General discussion*.

Again, we compared the test performance to chance (50%) for each condition separately. The JOL condition only significantly exceeded chance performance on Baseline trials, t(56) = 4.15, *p* < 0.001, and performed significantly worse than chance on the Cross-Mapped test trials (indicating a preference towards featural-responding), t(56) = 2.79, *p* = 0.007 (all other conditions *p* > 0.05). In contrast, the Control condition significantly exceeded chance performance in all conditions (all *p* < 0.05) except Cross-Mapped trials, t(62) = 0.32, *p* = 0.750. This differs from Experiment 1 where control participants demonstrated evidence of a rule-learning preference on Cross-Mapped test trials (and chance performance on Featural trials). This may indicate control participants demonstrated learning of both features and rules.

The results replicate the key findings of Experiment 1 and provide further support for the conservative strategy-shift account. When JOL participants were not given a hint they showed poorer rule discovery, but when they were directed towards a particular strategy, no reactivity was observed. Importantly, we found no evidence that JOLs could enhance rule learning when participants were instructed to attend to rule-relevant dimension of the stimulus, suggesting that JOLs do not merely reinforce participants initially adopted strategy but can instead cause a shift in strategies away from rule discovery. We also observe negative reactivity on the baseline test trials when a relational hint was provided, notably there was no evidence of negative reactivity on baseline test trials in either the no hint condition or the featural hint condition (nor was there in Experiment 1). While we cannot offer a comprehensive explanation of this effect (or rule out the possibility of a Type 1 error), it may be that, in combination with the rule hint, JOLs caused participants to be less flexible in the learning strategies (e.g., more narrowly focusing on rule learning) which ultimately produced poorer learning. However, this explanation is only speculative, and replication is required. Given the focus of the current exploration is on reactivity and rule discovery (rather than learning more generally), we leave further probing of this effect to subsequent investigations.

## Experiment 3

The first two experiments show that JOLs can impair relational rule discovery. We argue that JOLs promote viable performance orientated strategies in the environment, which ultimately impairs the learning of less obvious relational structures. We showed in Experiment 2 when we orient participants towards either a featural or relational strategy then there is less evidence of reactivity (though we did observe an effect on Baseline test trials). In Experiment 3, we further consider the predictions of the conservative strategy-shift account by examining the effect of JOLs on relational rule discovery when only the relational rule and not the surface features are predictive of category membership. The conservative strategy-shift account would suggest that there is no viable “shift” in strategies that participants can make in such a context and thus we should not observe reactivity.

### Method

#### Participants

Participants were recruited in the same manner as the prior experiments. Unlike the previous experiments, only baseline test trials were collected (as there was no correlation between features and category membership). As such, we collected data from 200 participants (50.5% female; M_age_ = 40.04 years, SD = 14.77 years) based on a power analysis for an independent-samples t-test (d = 0.4, power = 0.8). Ninety-five participants completed the task with JOLs and 105 participants completed the task in the control condition.

#### Materials and procedure

The materials and procedure were the same as Experiment 1 except there was now no relationship between the color of a stimulus and its category membership. The colors of the squares used in the training phase were sampled from a probability distribution (or red, green, yellow, blue) with a probability of 25% of each color.

As there was no feature to learn we used a simplified test phase procedure where participants just completed eight test trials that were defined by the same parameters as training (all stimuli are functionally equivalent to the relation test trials in the previous experiments).

### Results

#### Training data

**Fig. 6:**
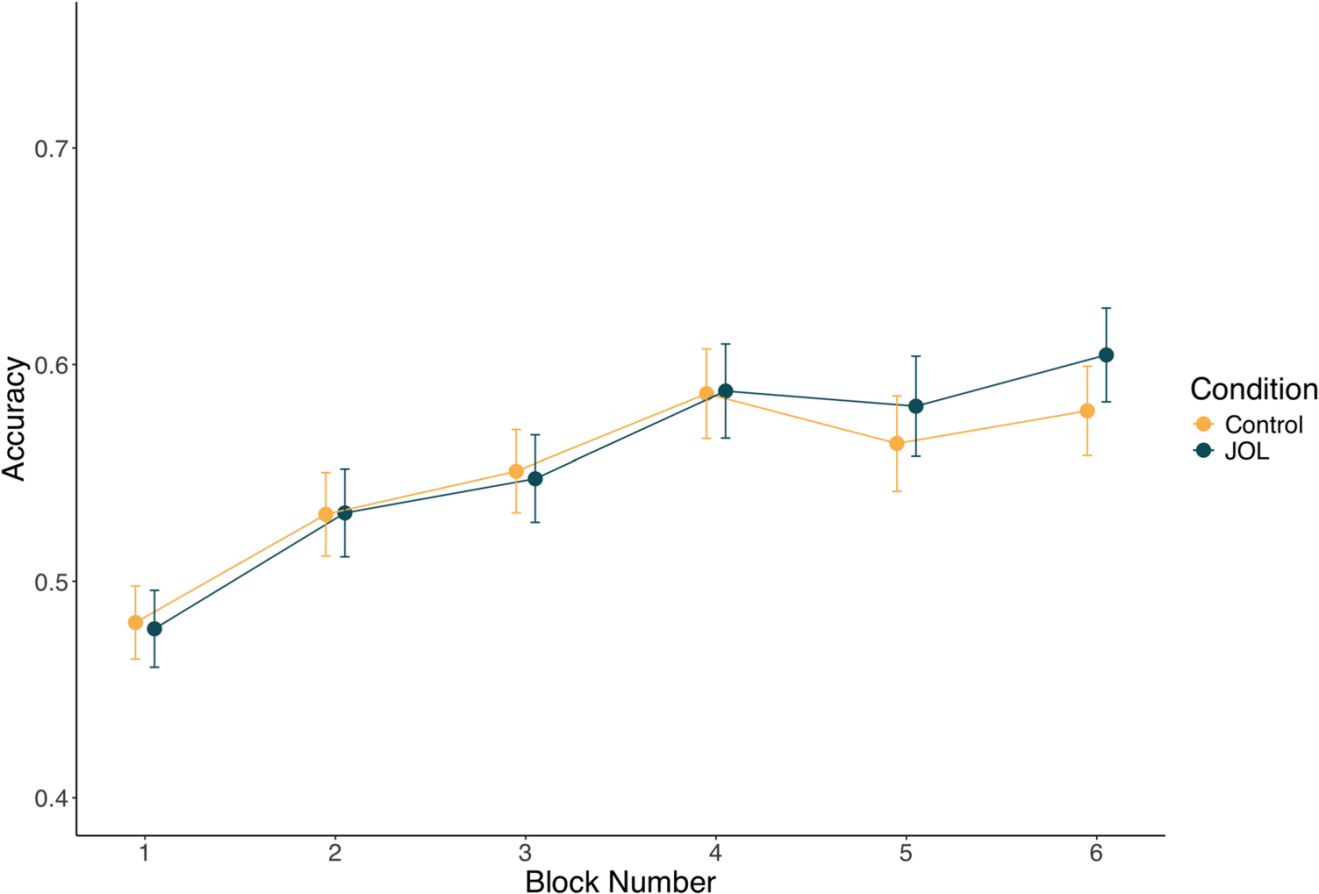
Experiment 3 training data from across the six blocks as a function of experimental condition. Error bars represent one standard error of the mean

Analyzing the training data using the same multilevel model as Experiment 1, we found a significant main effect of training block (β = 0.15, *p* < 0.001) (see Fig. 6). Neither the main effect of condition nor the interaction between condition and block number were significant.

#### Test data

Test data were analyzed using a simple t-test. There was no significant difference in test performance between the JOL (M = 0.56, SD = 0.27) and control (M = 0.56, SD = 0.25) conditions, t(198) = 0.031, *p* = 0.978, *d* = 0.004. When group means were compared to 50%, both conditions performed significantly above chance (both *p* < 0.05).

## General discussion

Recent evidence has shown that JOLs can affect memorization (Double et al., 2018; Mitchum et al., 2016; Soderstrom et al., 2015). Here, we extend these findings into the field of relational rule learning. In three experiments, we evaluated whether JOLs affected participants’ ability to discover a relational rule in a category-learning paradigm. In Experiments 1 and 2 we found that when both a relational rule and surface features of the stimuli were diagnostic of category membership, JOL impaired relational rule discovery. In contrast, we found that relational rule discovery was unaffected both when participants were orientated towards a particular strategy through instructional hints and when only the relational rule was diagnostic of category membership. Taken together these results support the notion that, when there are multiple effective strategies, and participants are not directed to use a particular one, JOLs will prompt them to rely on the more expedient surface strategy rather than discovering the more subtle rule-based strategy, which is often the strategy we wish to encourage in real-world learners.

Relational rule discovery is one of the most important aspects of learning and is the foundation for how students learn new concepts and the causal structure of the world (Goldwater et al., 2018). Relational rule discovery also requires many distinct cognitive processes compared to memorization such as abstraction, inference, induction, and deduction (Murphy, 2002). While there are ample studies showing a positive effect of JOLs on memorization (Double et al., 2018), we know little about how JOLs affect learning that relies on this more expansive set of cognitive processes. Importantly, while we observed poorer relational rule discovery, we make a distinction based on our findings between impaired rule-learning and strategy shifts away from rule learning. If JOLs impaired the cognitive processes involved in relational rule discovery (e.g. induction) we should have observed negative reactivity in Experiment 3 and in the relation hint conditions in Experiment 2. Instead, we found that participants shift their strategies away from rule discovery only when other viable strategies exist. We argue that this distinction supports the notion that, in this case, reactivity is less driven by a direct change in cognitive processes such as abstraction and inference, but rather a change in the metacognitive control processes involved in strategy selection. Indeed, these findings fit with the majority of metacognitive models that posit a close link between monitoring and control processes for adaptive goal-directed behavior (Carlebach & Yeung, 2020; Nelson & Narens, 1994).

Several studies have argued that reactivity can, in part, be explained by metacognitive ratings causing participants to shift to more conservative strategies (Double & Birney, 2017, 2018; Mitchum et al., 2016). This is also in keeping with several studies using word-list paradigms which have found that JOLs result in participants adopting strategies that prioritize the processing of item-specific features, which can come at the cost of inter-item relational processing (Senkova & Otani, 2021; Zhao et al., 2023a, 2023b). Crucially, this strategy shift does not necessarily mean that learners ignore every relation among items; instead, it means they may be more likely to rely on simple frequency tallies of intrinsic cues that can be encoded one stimulus at a time. In our paradigm, color frequency fits that description: a participant can note “this pattern is mostly red/green” and, across trials, accumulate a running count without ever comparing the internal structure of the lines. By contrast, discovering the monotonicity rule demands a configural comparison of line lengths within each stimulus and an abstraction of that relation across stimuli – a higher-order operation shown to be sensitive to working-memory load and attentional competition (Goldwater & Schalk, 2016). We therefore propose that JOLs bias attention toward the readily coded color cue, leaving fewer resources for the more computation-heavy relational abstraction; this predicts selective impairment of rule-based learning while leaving (or even slightly helping) feature-based performance.

The current findings would seem to support this conservative strategy-shift account by suggesting that when a more obvious strategy is available (e.g., a strategy that focuses on item-specific processing), participants who make JOLs are less inclined to discover relational rules. JOLs necessitate that participants make a prediction about their performance on a future test. This repeated requirement to self-assess appears to prioritize more conservative strategies and narrow participants goals to their immediate performance. The idea that what is measured is targeted, often at the expense of other outcomes, is well established across a broad range of domains (Elton, 2004). The notion that eliciting metacognitive ratings produces more conservative performance has also been borne out by drift–diffusion modeling. In what they refer to as the conservatism hypothesis, Li et al. (2023) found that eliciting confidence ratings increase participants’ response thresholds (they require greater confidence before responding), suggesting they become more conservative in their approach to cognitive tasks. Further work is needed to identify exactly why JOLs might prompt more conservative performance-oriented strategy use. For example, is it the repeated requirement to self-assess or the exact phrasing of JOLs? There is, for example, evidence that reactivity effects depend on the wording of the rating (Double & Birney, 2019a). Furthermore, several studies have argued that other mechanisms provide a better explanation of reactivity in paired-associates paradigms (e.g., Rivers et al., 2021; Tekin & Roediger, 2020).

Our results refine and extend the pattern reported by Double et al. (2025). In their three-group design, only confidence ratings reduced rule-based responding, whereas JOLs produced performance indistinguishable from a no-rating control. The present experiments show that JOLs are not intrinsically innocuous: when an easily memorized surface cue (color frequency) competes with the relational rule, and when the JOL is framed as an all-items accuracy prediction, the same selective impairment emerges. Taken together, the two data sets delineate clear boundary conditions for metacognitive reactivity. A prompt disrupts abstraction whenever it (a) continually draws attention to upcoming success or failure and (b) leaves open a quicker, lower-level route to that success; which specific prompt (confidence vs. JOL) fulfils those conditions depends on the wording of the judgment and the structure of the learning environment. The convergence supports the broader conservative strategy-shift account, in which on-line self-assessment nudges learners toward the most readily rewarded strategy – feature memorisation here, slower but surer responding in other paradigms – at the expense of deeper relational learning. Practically, this means that classroom or laboratory interventions that ask students to “rate how well you’ll do” may backfire whenever a superficial cue can deliver short-term correctness; careful prompt wording and task design are therefore critical if JOLs are to be used as a pedagogical tool rather than a distraction.

Recognizing common patterns in the relations among objects is critical for education and knowledge transfer. Often surface features of learning materials correlate with deeper relational structures (Goldwater et al., 2018), and it is crucial for learning and transfer that students can shift focus to the relational aspect of the category and foster transfer. For instance, McDaniel et al. (2018) found that students who abstract rules demonstrate an advantage on transfer questions in a chemistry exam compared to students who focus on memorization, while there was no difference on exam problems that were very similar to class or homework. Metacognitive judgments are said to be an important driver of students’ decisions about which information to attend to (Butler & Winne, 1995; Metcalfe & Kornell, 2005). However, our results suggest that JOLs are likely to be counterproductive when the goal is to have students attend to the deeper relational structure of learning materials. It is unclear whether the effect of explicit JOLs in terms of directing attention away from relational structures runs counter to how participants spontaneously monitor their performance or, in fact, amplifies learners’ normal metacognitive beliefs. Indeed, experiments using this same category paradigms without JOLs has shown that most participants fail to discover the rule, suggesting that even without explicit metacognitive judgments most participants do not adopt a metacognitive strategy that facilitates rule discovery (Goldwater et al., 2018).

While the current studies suggest that JOLs negatively affect rule discovery, there are several limitations to note. First, there are many other viable reasons why JOLs can be reactive (e.g., see Soderstrom et al., 2015, for a discussion of the cue-strengthening hypothesis). Our results do not speak to the viability of these other reactivity mechanisms as they are not mutually exclusive and, indeed, some of these other mechanisms may have a bearing on our findings. We limited ourselves here to a discussion of the conservative strategy-shift account of reactivity because it provides a parsimonious explanation of our findings, but further work looking at the boundary conditions and other contextual effects is needed to fully describe the mechanisms at play when JOLs are elicited. Second, the stimuli used in the current study are by design relatively constrained and further effort is needed to examine how JOLs affect learning of more educationally relevant materials. For instance, Ariel et al. (2021) found that instructing students to make JOLs did not improve comprehension of an educational textbook. Given that rule discovery is an important classroom analogue (Goldwater & Schalk, 2016), it is important that these findings are further expanded and replicated with educationally relevant materials.

Furthermore, JOLs typically assess the likelihood of remembering a recently studied piece of information during a later test (e.g., “Please indicate the chances that you will remember this word at test?”). In the current experiments, participants were asked: “Please rate the likelihood that you will correctly indicate whether items are Blickets or Snargs on a later test.” Unlike traditional JOLs, which apply to specific items, this prompt refers to any potential test item. This wording aligns with how JOLs have been used in some previous category learning studies (Lee & Ha, 2019). However, the exact phrasing of JOLs may be an important factor to consider. Because the JOLs in this study are not item-specific but instead apply to all possible test items, this distinction may influence the observed reactivity effects. For example, the wording of the JOL may imply the goal that a participant should adopt and thus influence the strategy they opt to utilize.

The current study is the first to examine the effect of JOLs on relational rule-based discovery. The results fit with the idea that JOLs prompt participants to use more obvious performance-orientated strategies, supporting a goal-shift mechanism as a part of reactivity. The current findings are somewhat discouraging for educators who would use such judgments in a classroom. While there is ample evidence that JOLs can improve memorization, this may come at the cost of the deeper learning that is often the ultimate goal of education.

## Supplementary Information

Below is the link to the electronic supplementary material.

Supplementary file1 (DOCX 274 KB)

## Data Availability

Data and materials are available in the Open Science Framework (https://osf.io/t4fcr).

## Code availability

Code is available in the Open Science Framework (https://osf.io/t4fcr/).

## References

[CR1] Ariel, R., Karpicke, J. D., Witherby, A. E., & Tauber, S. K. (2021). Do judgments of learning directly enhance learning of educational materials? *Educational Psychology Review,**33*, 693–712.

[CR2] Bassok, M., Chase, V. M., & Martin, S. A. (1998). Adding apples and oranges: Alignment of semantic and formal knowledge. *Cognitive Psychology,**35*, 99–134. 9570897 10.1006/cogp.1998.0675

[CR3] Butler, D. L., & Winne, P. H. (1995). Feedback and self-regulated learning: A theoretical synthesis. *Review of Educational Research,**65*(3), 245–281.

[CR4] Carlebach, N., & Yeung, N. (2020). Subjective confidence acts as an internal cost-benefit factor when choosing between tasks. *Journal of Experimental Psychology: Human Perception and Performance,**46*(7), 729–748. 32271082 10.1037/xhp0000747

[CR5] Davis, T., Goldwater, M., & Giron, J. (2017). From concrete examples to abstract relations: The rostrolateral prefrontal cortex integrates novel examples into relational categories. *Cerebral Cortex,**27*(4), 2652–2670. 27130661 10.1093/cercor/bhw099

[CR6] Double, K. S. (2023). Do Judgments of Learning Impair Recall When Uninformative Cues Are Salient? *Journal of Intelligence,**11*(10), 203. 37888435 10.3390/jintelligence11100203 PMC10607944

[CR7] Double, K. S., & Birney, D. P. (2017). *The interplay between self-evaluation, goal orientation, and self-efficacy on performance and learning* Proceedings of the 39th Annual Conference of the Cognitive Science Society.

[CR8] Double, K. S., & Birney, D. P. (2018). Reactivity to confidence ratings in older individuals performing the latin square task. *Metacognition and Learning,**13*(3), 309–326.

[CR10] Double, K. S., & Birney, D. P. (2019a). Do confidence ratings prime confidence? *Psychonomic Bulletin & Review,**26*, 1035–1042. 10.3758/s13423-018-1553-3 PMC6557865 30632106

[CR9] Double, K. S., & Birney, D. P. (2019b). Reactivity to measures of metacognition. *Frontiers in Psychology,**10*, 2755. 10.3389/fpsyg.2019.02755 PMC6908488 31866919

[CR11] Double, K. S., Birney, D. P., & Walker, S. A. (2018). A meta-analysis and systematic review of reactivity to judgements of learning. *Memory,**26*(6), 741–750. 29161973 10.1080/09658211.2017.1404111

[CR12] Double, K. S., Goldwater, M. B., & Birney, D. P. (2025). Reactivity to confidence ratings: Evidence of impaired rule-learning. *Metacognition and Learning,**20*(1), 8.

[CR13] Doyle, M. E., & Hourihan, K. L. (2016). Metacognitive monitoring during category learning: How success affects future behaviour. *Memory,**24*(9), 1197–1207. 26377626 10.1080/09658211.2015.1086805

[CR14] Elton, L. (2004). Goodhart’s Law and performance indicators in higher education. *Evaluation & Research in Education,**18*(1–2), 120–128.

[CR15] Emmons, N., Lees, K., & Kelemen, D. (2018). Young children’s near and far transfer of the basic theory of natural selection: An analogical storybook intervention. *Journal of Research in Science Teaching,**55*(3), 321–347.

[CR16] Goldwater, M. B., Don, H. J., Krusche, M. J., & Livesey, E. J. (2018). Relational discovery in category learning. *Journal of Experimental Psychology: General,**147*(1), 1. 29309195 10.1037/xge0000387

[CR17] Goldwater, M. B., & Schalk, L. (2016). Relational categories as a bridge between cognitive and educational research. *Psychological Bulletin,**142*(7), 729–757. 26950007 10.1037/bul0000043

[CR18] Halamish, V., & Undorf, M. (2023). Why do judgments of learning modify memory? Evidence from identical pairs and relatedness judgments. *Journal of Experimental Psychology: Learning, Memory, and Cognition,**49*(4), 547. 36006723 10.1037/xlm0001174

[CR19] Janes, J. L., Rivers, M. L., & Dunlosky, J. (2018). The influence of making judgments of learning on memory performance: Positive, negative, or both? *Psychonomic Bulletin & Review*, 1–9. 10.3758/s13423-018-1463-4 29611141

[CR20] Kaya, S., & Mulligan, N. W. (2024). Metamemory judgments and design effects: Judgment of learning (JOL) reactivity in free recall is affected by study list structure. *Memory & Cognition*, 1–15. 10.3758/s13421-024-01638-0 39312124

[CR21] Koriat, A. (2007). Metacognition and consciousness. In P. D. Zelazo, M. Moscovitch, & E. Thompson (Eds.), *The Cambridge Handbook of Consciousness* (pp. 289–325). Cambridge University Press.

[CR22] Kurtz, K. J., Boukrina, O., & Gentner, D. (2013). Comparison promotes learning and transfer of relational categories. *Journal of Experimental Psychology: Learning, Memory, and Cognition,**39*(4), 1303. 23421515 10.1037/a0031847

[CR23] Lee, H. S., & Ha, H. (2019). Metacognitive judgments of prior material facilitate the learning of new material: The forward effect of metacognitive judgments in inductive learning. *Journal of Educational Psychology,**111*(7), 1189.

[CR24] Li, B., Hu, X., Shanks, D. R., Su, N., Zhao, W., Meng, L., ... & Yang, C. (2023). Confidence ratings increase response thresholds in decision making. *Psychonomic Bulletin & Review*, 1–10. 10.3758/s13423-023-02380-5 37803229

[CR25] Li, B., Zhao, W., Zheng, J., Hu, X., Su, N., Fan, T., Yin, Y., Liu, M., Yang, C., & Luo, L. (2021). Soliciting judgments of forgetting reactively enhances memory as well as making judgments of learning: Empirical and meta-analytic tests. *Memory & Cognition*, 1–17. 10.3758/s13421-021-01258-y 34855150

[CR26] Maxwell, N. P., & Huff, M. J. (2022). Reactivity from judgments of learning is not only due to memory forecasting: Evidence from associative memory and frequency judgments. *Metacognition and Learning,**17*(2), 589–625. 35505852 10.1007/s11409-022-09301-2 PMC9051498

[CR27] McDaniel, M. A., Cahill, M. J., Frey, R. F., Rauch, M., Doele, J., Ruvolo, D., & Daschbach, M. M. (2018). Individual differences in learning exemplars versus abstracting rules: Associations with exam performance in college science. *Journal of Applied Research in Memory and Cognition,**7*(2), 241–251.

[CR28] Metcalfe, J., & Kornell, N. (2005). A region of proximal learning model of study time allocation. *Journal of Memory and Language,**52*(4), 463–477.

[CR29] Mitchum, A. L., Kelley, C. M., & Fox, M. C. (2016). When asking the question changes the ultimate answer: Metamemory judgments change memory. *Journal of Experimental Psychology: General,**145*(2), 200. 27045282 10.1037/a0039923

[CR30] Murphy, G. L. (2002). *The big book of concepts.* MIT Press.

[CR31] Myers, S. J., Rhodes, M. G., & Hausman, H. E. (2020). Judgments of learning (JOLs) selectively improve memory depending on the type of test. *Memory & Cognition,**48*, 745–758. 32124334 10.3758/s13421-020-01025-5

[CR32] Nelson, T. O., & Narens, L. (1994). Why investigate metacognition? In J. Metcalfe & A. P. Shimamura (Eds.), *Metacognition: Knowing about knowing* (pp. 1–25). The MIT Press.

[CR33] Resnick, L. B. (2010). Nested learning systems for the thinking curriculum. *Educational Researcher,**39*(3), 183–197.

[CR34] Rivers, M. L., Janes, J. L., & Dunlosky, J. (2021). Investigating memory reactivity with a within-participant manipulation of judgments of learning: Support for the cue-strengthening hypothesis. *Memory,**29*(10), 1342–1353. 34635008 10.1080/09658211.2021.1985143

[CR35] Rohrer, D., Dedrick, R. F., & Burgess, K. (2014). The benefit of interleaved mathematics practice is not limited to superficially similar kinds of problems. *Psychonomic Bulletin & Review,**21*, 1323–1330. 24578089 10.3758/s13423-014-0588-3

[CR36] Senkova, O., & Otani, H. (2021). Making judgments of learning enhances memory by inducing item-specific processing. *Memory & Cognition,**49*, 955–967. 33398785 10.3758/s13421-020-01133-2

[CR37] Shi, A., Xu, C., Zhao, W., Shanks, D. R., Hu, X., Luo, L., & Yang, C. (2023). Judgments of learning reactively facilitate visual memory by enhancing learning engagement. *Psychonomic Bulletin & Review,**30*(2), 676–687. 36109421 10.3758/s13423-022-02174-1

[CR38] Soderstrom, N. C., Clark, C. T., Halamish, V., & Bjork, E. L. (2015). Judgments of learning as memory modifiers. *Journal of Experimental Psychology: Learning, Memory, and Cognition,**41*(2), 553. 25528101 10.1037/a0038388

[CR39] Tauber, S. K., & Witherby, A. E. (2019). Do judgments of learning modify older adults’ actual learning? *Psychology and Aging,**34*(6), 836. 31259565 10.1037/pag0000376

[CR40] Tekin, E., & Roediger III, H. L. (2020). Reactivity of judgments of learning in a levels-of-processing paradigm. *Zeitschrift für Psychologie*.

[CR41] Undorf, M., Ingendahl, F., & Halamish, V. (2024). Making Judgments of Learning Either Enhances or Impairs Memory: Evidence From 17 Experiments With Related and Unrelated Word Pairs. *Collabra: Psychology*, *10*(1).

[CR42] Veenman, M. V. J., Van Hout-Wolters, B. H. A. M., & Afflerbach, P. (2006). Metacognition and learning: Conceptual and methodological considerations. *Metacognition and Learning,**1*(1), 3–14.

[CR43] Wang, X., Liu, X., Chen, L., Feng, K., Ye, Q., & Zhu, H. (2023). The Forward Effect of Delayed Judgments of Learning Is Influenced by Difficulty in Memory and Category Learning. *Journal of Intelligence,**11*(6), 101. 37367503 10.3390/jintelligence11060101 PMC10299460

[CR44] Witherby, A. E., & Tauber, S. K. (2017). The Influence of Judgments of Learning on Long-Term Learning and Short-Term Performance. *Journal of Applied Research in Memory and Cognition,**6*(4), 496–503.

[CR45] Yang, H., Cai, Y., Liu, Q., Zhao, X., Wang, Q., Chen, C., & Xue, G. (2015). Differential neural correlates underlie judgment of learning and subsequent memory performance. *Frontiers in Psychology,**6*, 1699. 26617540 10.3389/fpsyg.2015.01699 PMC4637415

[CR46] Zhao, W., Li, B., Shanks, D. R., Zhao, W., Zheng, J., Hu, X., Su, N., Fan, T., Yin, Y., & Luo, L. (2022). When judging what you know changes what you really know: Soliciting metamemory judgments reactively enhances children’s learning. *Child Development,**93*(2), 405–417. 34655225 10.1111/cdev.13689

[CR47] Zhao, W., Li, J., Shanks, D. R., Li, B., Hu, X., Yang, C., & Luo, L. (2023a). Metamemory judgments have dissociable reactivity effects on item and interitem relational memory. *Journal of Experimental Psychology: Learning, Memory, and Cognition,**49*(4), 557. 36848046 10.1037/xlm0001160

[CR48] Zhao, W., Xu, M., Xu, C., Li, B., Hu, X., Yang, C., & Luo, L. (2023b). Judgments of Learning Following Retrieval Practice Produce Minimal Reactivity Effect on Learning of Education-Related Materials. *Journal of Intelligence,**11*(10), 190. 37888422 10.3390/jintelligence11100190 PMC10607076

[CR49] Zimmerman, B. J., & Moylan, A. R. (2009). Self-regulation: Where metacognition and motivation intersect. In *Handbook of metacognition in education* (pp. 299–315). Routledge.

