# Supplementary material for: Judgments of learning impair rule-based discovery
Source: Mem Cognit. 2025 Jun 4;54(1):45–58. doi: 10.3758/s13421-025-01737-6 (PMC12864244; doi:10.3758/s13421-025-01737-6)
Supplement: Supplementary file 1 — Supplementary file1 (DOCX 274 KB) [file 13421_2025_1737_MOESM1_ESM.docx]

**Supplementary Materials**

**Experiment 1**

Average JOL across the training phase was 50.07 (SD = 25.28). JOLs drawn from the six training blocks was subjected to a multilevel model. JOLs increased significantly across training (β = .15, *p* < .001), see Figure S1.


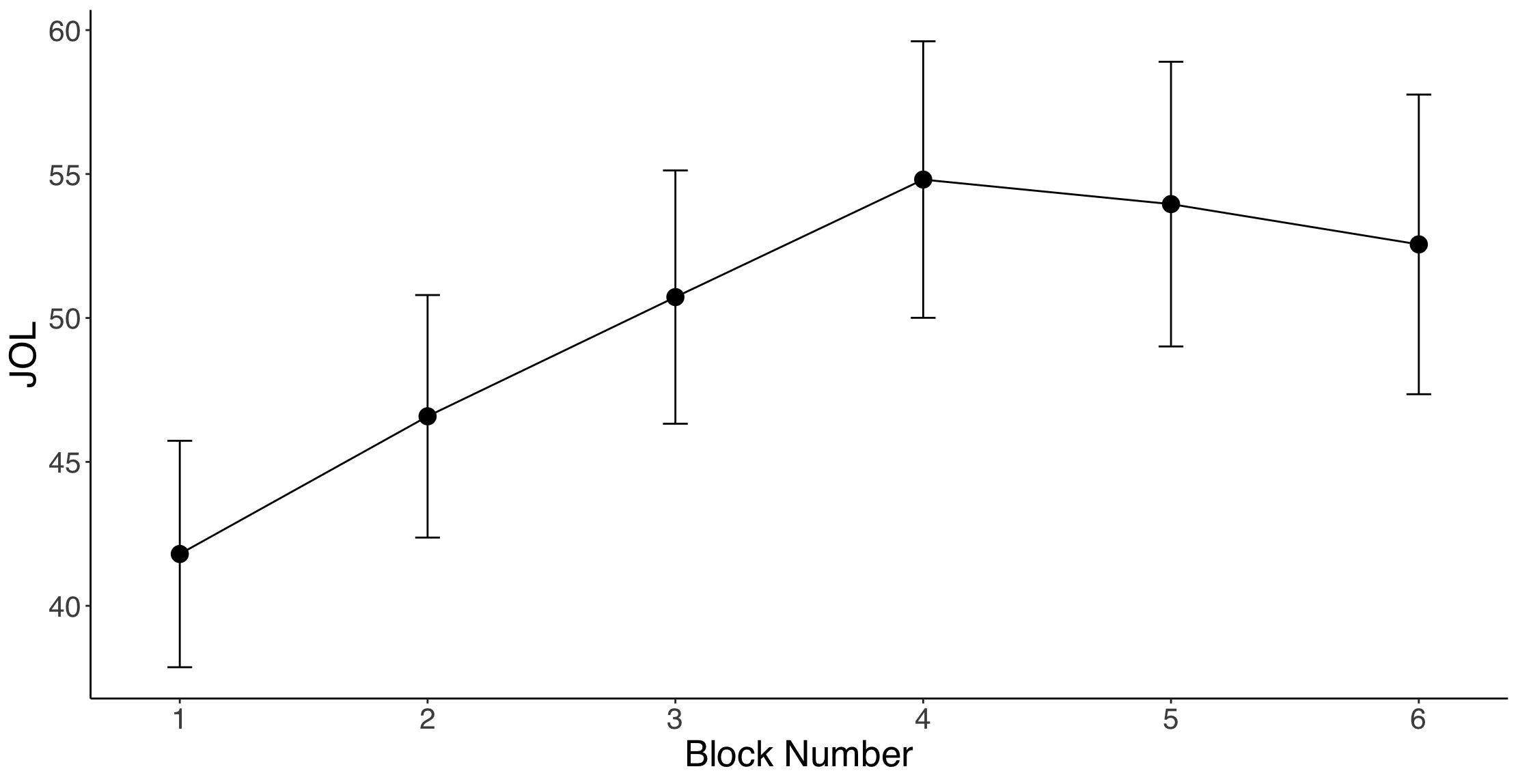


*Figure S1.* JOLs as a function of training block. Error bars are +1 standard error of the mean

**Experiment 2**

Average JOL across the training phase was 38.84 (SD = 22.91). JOLs drawn from the six training blocks was subjected to a multilevel model with the interaction between hint condition and block number included. JOLs increased significantly across training (β = .15, *p* < .001) but the main effect of hint condition and the interaction between hint condition and block number were not significant, see Figure S2.


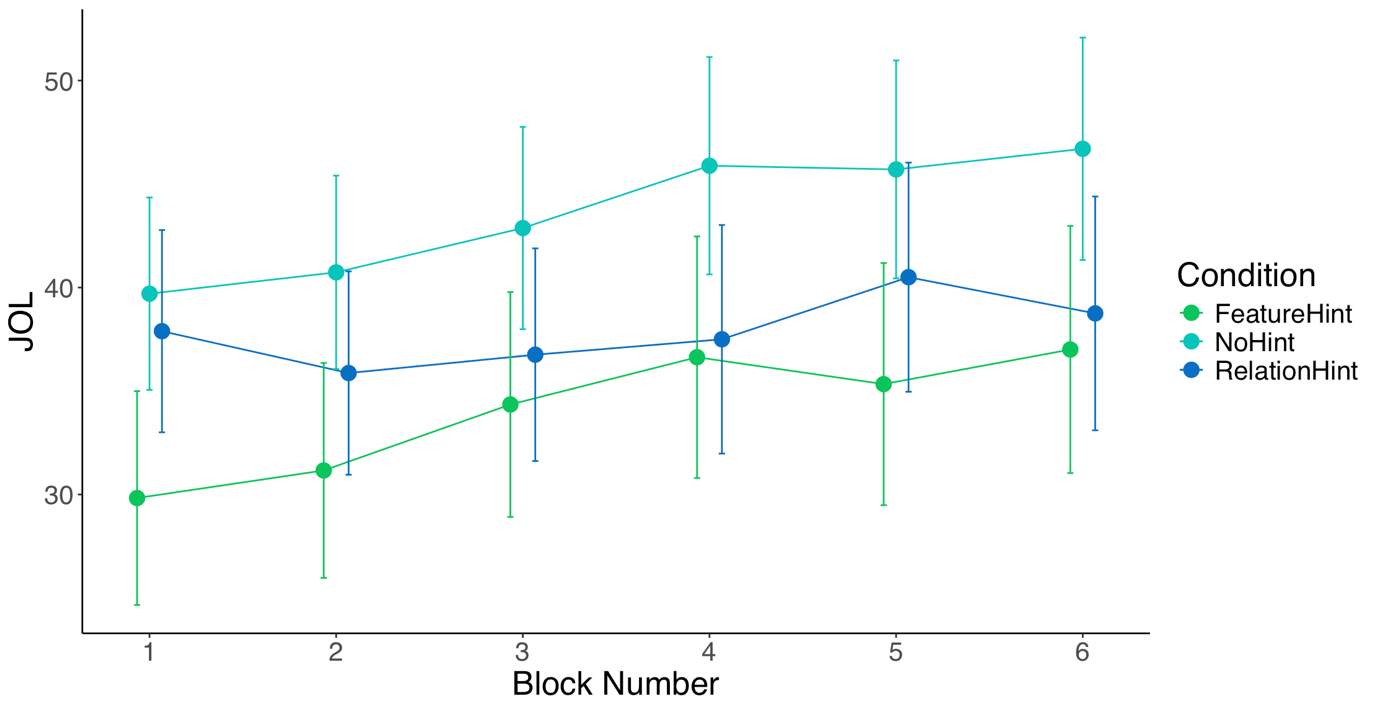


*Figure S2.* JOLs as a function of training block. Error bars are +1 standard error of the

**Experiment 3**

Average JOL across the training phase was 41.08 (SD = 27.14). JOLs drawn from the six training blocks was subjected to a multilevel model. JOLs increased significantly across training (β = .13, *p* < .001), see Figure S3.


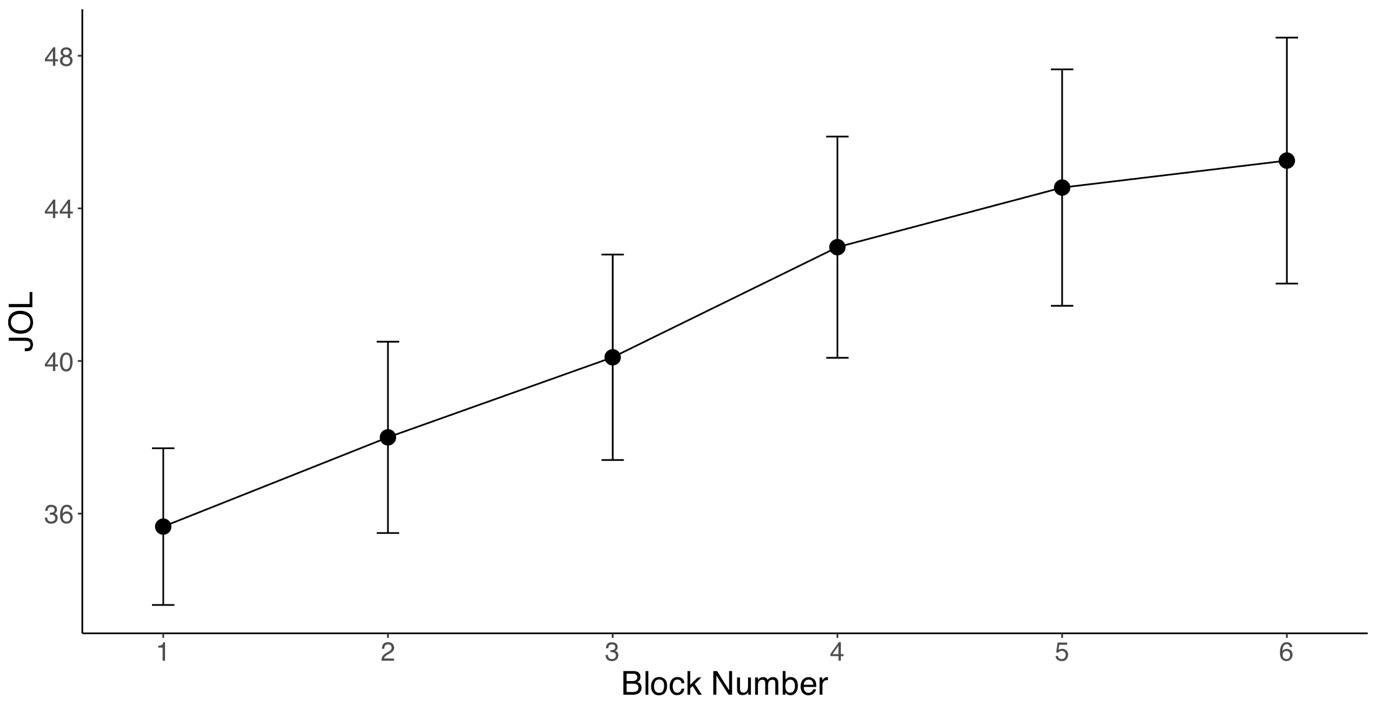


*Figure S3.* JOLs as a function of training block. Error bars are +1 standard error of the mean
